# An electron microscopic study of the archaeal feast/famine regulatory protein 5. Fourier filtration of images

**Published:** 2004-04-01

**Authors:** Sanae A. Ishijima, Lester Clowney, Masashi Suzuki

**Affiliations:** *) National Institute of Advanced Industrial Science and Technology (AIST), AIST Tsukuba Center 6-10, Higashi, 1-1-1, Tsukuba, Ibaraki 305-8566, Japan; **) Japan Science and Technology Agency (JST), Core Research for Evolutionary Science and Technology (CREST), Honmachi, 4-1-18, Kawaguchi Center Building, Kawaguchi, Saitama 332-0012, Japan

**Keywords:** Cryo-electron microscopy, Fourier analysis, hyper-thermophiles, image processing, protein electron microscopy

## Abstract

Microcrystals of the feast/famine regulatory protein (FFRP) pot0434017 (FL11) were prepared by sonicating larger crystals. Using the microcrystals a cryo-electron micrograph was obtained, which showed a hexagonal packing of cylinder-like assemblies of FL11. This micrograph was processed by selecting, in the Fourier space, spots reflecting the crystal lattice, thereby removing the noise. The microcrystal was not totally free from distortion, and cylinders in local clusters adopted slightly different orientations. Thus, 25 hexagonal units closest to the ideal, each containing a cylinder at the center surrounded by six others, were manually selected. The averaged image was further processed to yield a perfect six-fold symmetry. These processed images, and some of the original images too, show bridges connecting cylinders, each corresponding to two pairs of N-domains, protruding from the two cylinders and contacting between them in the X-ray structure.

## Introduction

While studying the structure and function of a group of bacterial transcription factors, the feast/famine regulatory proteins (FFRPs),1)–11) we have crystallized an FFRP, pot0434017 (FL11) from the hyper-thermophilic archaeon *Pyrococcus* sp. OT3.2),11) The crystals, when they are fully grown, reach the size [~0.4 mm] × [~0.4 mm] × [~0.2 mm]. Using these, the atomic 3D structure was determined by X-ray crystallography. 2) In the crystals cylinder-like assemblies of FL11 extend along the z axis, spanning ~0.2 mm, while packed with a hexagonal symmetry in the x-y plane of 0.4 mm × 0.4 mm.

These crystals, however, are too large for types of examinations using electron microscopes. Thus in our previous study,5) smaller crystals, still growing or remaining as such, were selectively used for a cryo-electron microscopic12) study. Among the images obtained were views of mono-layers of cylinders extending side by side along the z axis (here referred to as side views).

**Fig. 1:**
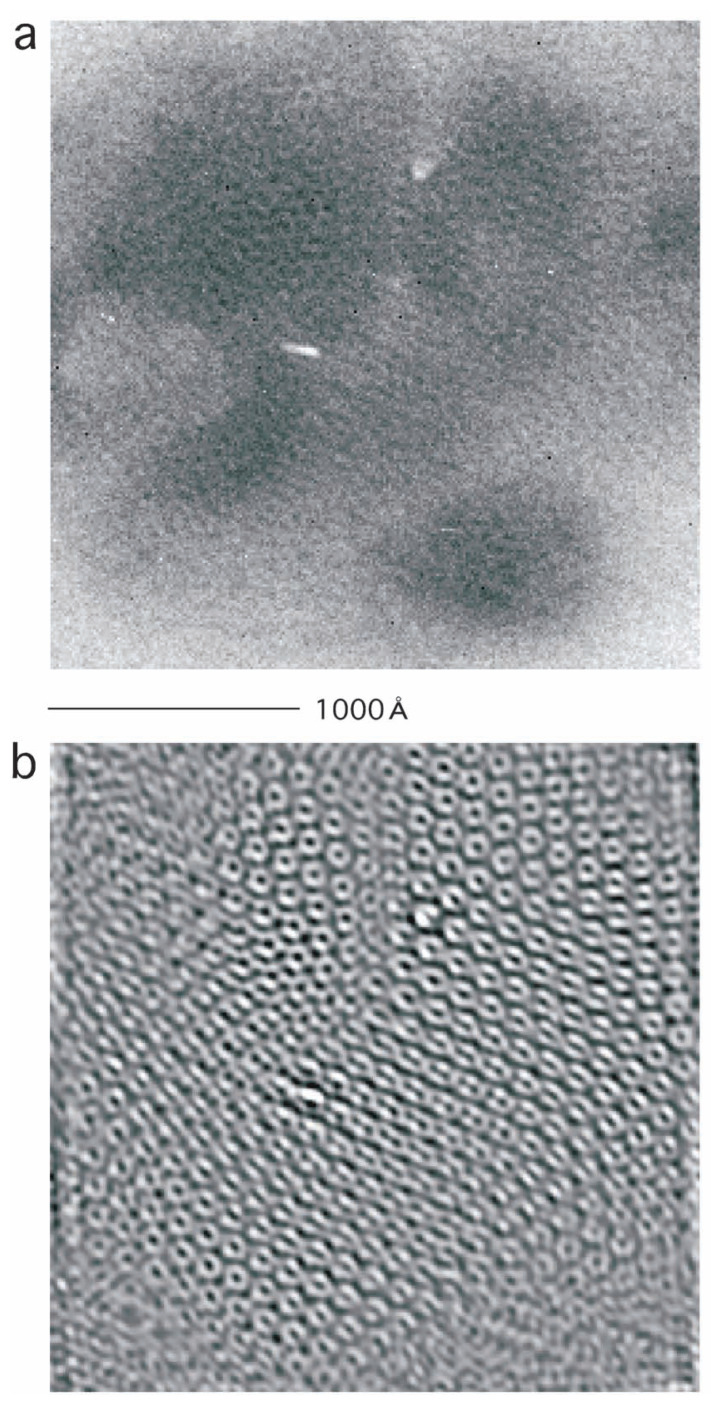
A quarter of the original (a, 512 × 512 pixels) and filtered (b) electron micrographs. The scale 1,000 Å is shown.

While making further attempts to obtain a better microcrystal, this time by sonicating larger crystals, we have obtained a top view, showing the hexagonal arrangement of cylinders, projected onto the x-y plane (Fig. 1a).

## Cryo-electron microscopy

The protein FL11 was expressed and purified as has been described, and its crystals were grown.2),11) A protein solution, ~20 mg/ml in 50 mM sodium phosphate buffer (pH = 8.0) containing 300 mM NaCl, was mixed with the same volume of the crystallization buffer, 0.1 M Tris-HCl buffer (pH = 8.5) containing 1.5 M ammonium sulfate and 12% (vol/vol) glycerol, and equilibrated with the same crystallization buffer in the reservoir. The mother solution, 5 μl, yielding crystals, was mixed with 45 μl of the doubly diluted crystallization solution in a 1.5 ml Eppendorf tube. While cooling in ice, the crystals were sonicated for 2 sec using a sonifer (type 250, Branson Ultrasonics Co., CT, USA, equipped with a micro-tip, and with the output control setting of 1).

A droplet, 4 μl, of the solution was placed on a holey carbon-coated copper grid (300 mesh, Electron Microscopy Sciences Co.). As before,2)–6) the specimen was kept in amorphous ice in order to minimize damage caused by dehydration and electron irradiation, i.e. the method of cryo-electron microscopy.12) The grid was quickly frozen in liquid ethane into an amorphous ice state, using a freezing apparatus (EM CPC, Leica). It was maintained at a near liquid nitrogen temperature using a holder (CT3500, Oxford), while an electron microscope (Tecnai F20, FEI) was operated at 200 KeV.

By irradiating electrons at a low dosage (~5 electrons/Å^2^), a micrograph was recorded at a magnification of 44.9 K, using a CCD (charge coupled device) camera (794IF, Gatan, 1,024 pixels × 1,024 pixels). In order to minimize chromatic aberration, electrons that had lost no energy (i.e. 200 KeV ± 10 eV) were selectively focused, using an energy filter (GIF200, Gatan). The CCD camera and the energy filter were operated using the Digital Micrograph package (Gatan).

## Fourier transform and filtration

A quarter (512 × 512 pixels) of the original electron micrograph obtained is shown in Fig. 1a. Cylinder-like assemblies of FL11 are visible there, each projected to a whitish “pencil cross-section” of the diameter ~100Å, whose hollow cavity at the center is visible as if it is the “lead”, i.e. the dark density. When more carefully observed, these “pencil cross-sections” are arranged with a hexagonal symmetry, each surrounded by six others.

**Fig. 2:**
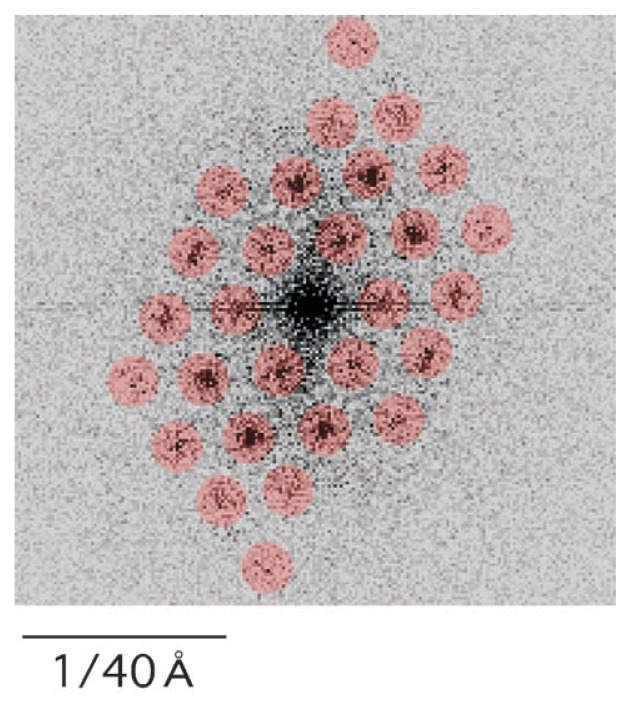
A Fourier transform of the electron micrograph (Fig. 1a). Spots used for inverse Fourier transform, thereby producing Fig. 1b, are marked in red.

The Fourier transform of this quarter was calculated using the Digital micrograph package (Fig. 2). Regular (i.e. hexagonal) spots reflecting the crystal lattice were selected (circled) with a masking size parameter of 5, and the inverse Fourier transform was calculated, producing a Fourier-filtered micrograph (Fig. 1b). It is clear that by this filtration some types of noise were removed, and some details of the cylinders were enhanced showing their hexagonal packing.

This filtration carried out computationally is, in fact, not different in its principle from experimental filtration of electron micrographs using an optical device, which was developed by Klug and DeRosie,13),14) in the days when the calculation power of computers was largely limited.

## Processing of images in the Fourier and real spaces

**Fig. 3:**
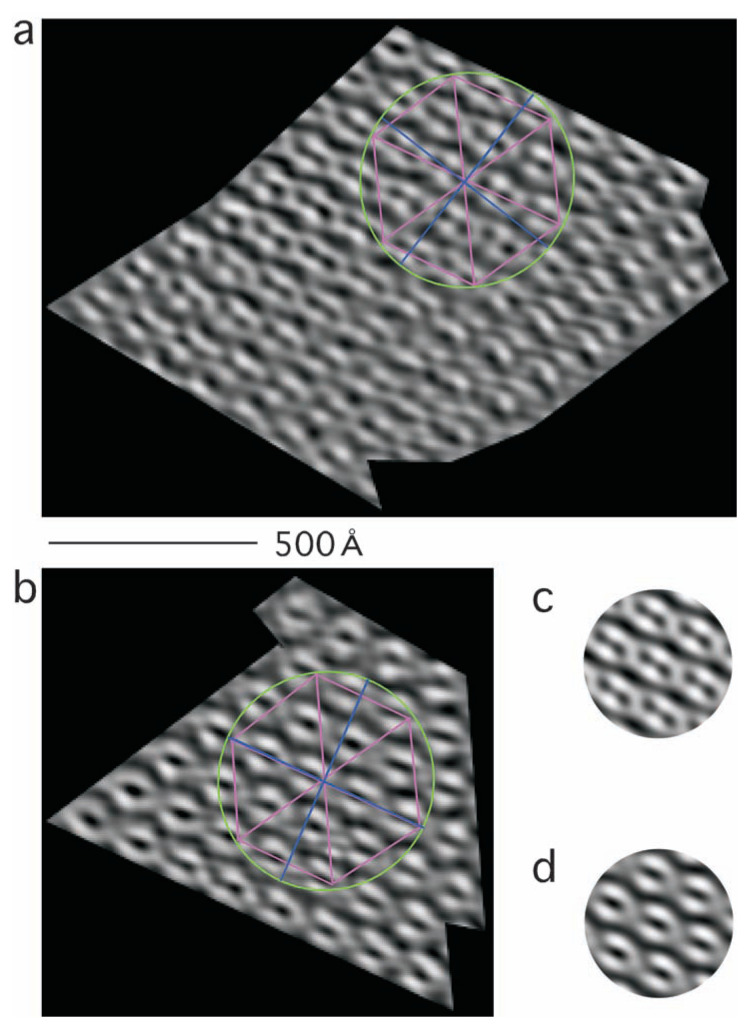
Two parts (a, b) of the filtered electron micrograph (Fig. 1b), and their pseudo-hexagonal units (c of a, and d of b). The scale 500 Å is shown. In (a) and (b) ellipsoids (green) circumscribe pseudo-hexagonal lattices (crimson). By comparing the two axes of each ellipsoid (blue), the tilt angle is determined (Fig. 5c). Of the pair, one axis (*d*cos***θ*** in Fig. 5c) is parallel to the tailing of the cylinders with the length *l*sin***θ*** (Fig. 5c).

The microcrystal, however, was not totally free from distortion, and local subpopulations of cylinders adopted slightly different tilts (see examples shown in Fig. 3a, b or averaged images, Fig. 3c, d). One way to select ideal projections of cylinders (i.e. those with less tilt) is to use smaller and more regular masks in the Fourier space by further limiting deviation from the ideal hexagonal packing. However, as will be discussed in later sections, some part of the protein assemblies are less ordered in the microcrystal, even if projected perfectly with no tilt. Use of smaller masks will remove these densities. Thus, instead, further processing was carried out in the real space as is described in the next section.

## Averaging and symmetrization of the images in the real space

**Fig. 4:**
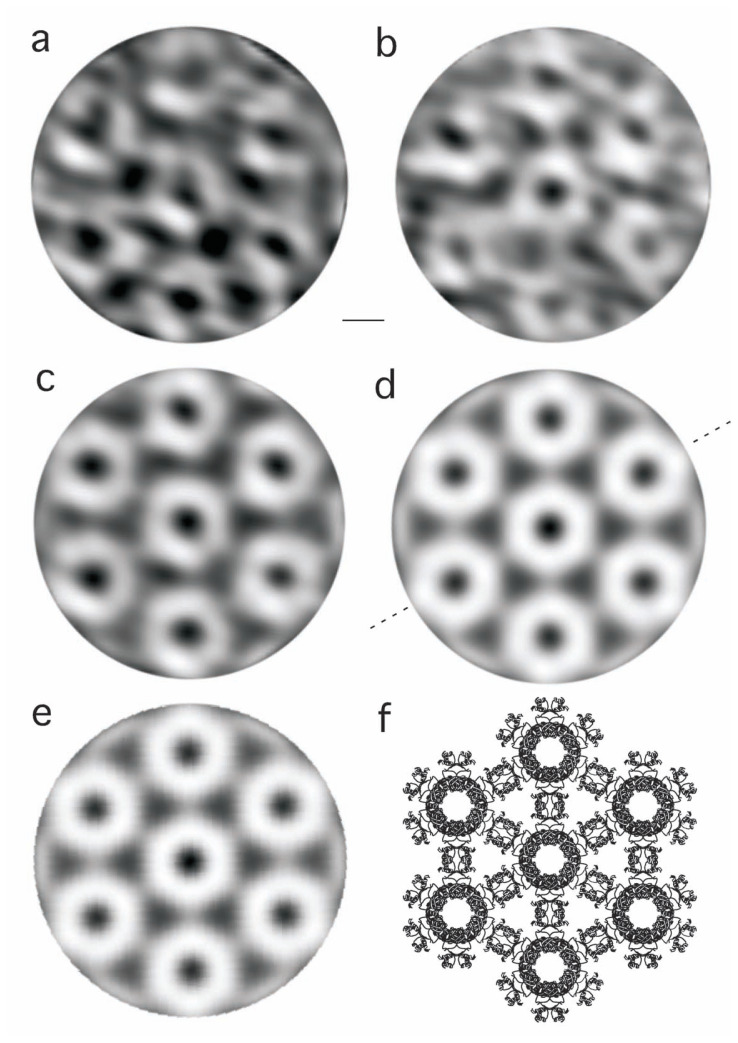
Two examples (a, b) of 25 hexagonal units selected from the Fourier-filtered micrograph (Fig. 1b) and used for averaging (c), followed by six-fold (d) and mirror (e) symmetrization, in comparison with a ribbon diagram of the X-ray structure (f). The mirror used for producing (e) from (d) is shown by a broken line in (d). The sale 50 Å is shown between (a) and (b).

Using the Fourier-filtered micrograph, 25 hexagonal units closest to the ideal, each containing a cylinder at the center surrounded by six others, were manually selected (Fig. 4a, b). These images were averaged, using the Proc2D program in the Eman package 15) (Fig. 4c).

The averaged image (Fig. 4c) was then processed to yield a perfect six-fold symmetry around the center of the hexagon (Fig. 4d). This symmetry relates the six cylinders surrounding the central one, as well as, inside the central cylinder, its six units forming a turn, each facing a particular cylinder outside, when projected along the helical axis. Here it is important to note that the focus of any electron microscope is deep, and thus an image obtained is not a section but a projection of the object.14)

It is known that the basic unit of any FFRP for assembling into various forms is a dimer,1) and thus the unit of FL11 cylinder too should have a two-fold symmetry. When this two-fold axis runs perpendicular to the helical axis of the cylinder, as it is known from the X-ray structure,2) a projection such as Fig. 4d should have a symmetry to the mirror placed by connecting, e.g., units 2 and 5 (shown by a broken line in Fig. 4d). The image (Fig. 4d) was averaged with its mirror image, yielding Fig. 4e.

## Comparison with the X-ray structure

Although the resolutions are not higher, the overall shapes and characteristics of the processed images (Fig. 4c–e) are in good agreement with a top view (Fig. 4f) of the atomic structure of FL11 determined by X-ray crystallography using larger crystals.2) Around the “lead” in the center, each “pencil” or the “doughnut” corresponds to the projection of the C-terminal halves of the protein molecules assembling with each other (Fig. 4f). This part of the EM images appears to be smoothly linked and well-defined. In fact, the temperature factor calculated for this part of the X-ray structure is small (i.e. well-ordered).

In contrast, while the two domains of FL11 have similar molecular weights, the density of the N-halves is less clear in the EM images. In the X-ray structure, the temperature factor of the N-halves is poorer, reflecting flexibility of the linker connecting the two domains in each monomer, and thus the position of the N-half. When assembled, two pairs of N-halves protrude from two dimers, each dimer in each cylinder, and contact with each other between the two cylinders (Fig. 4f), but these N-halves are, as a whole, less ordered. In the processed EM images (Fig. 4c–e) connections are found bridging cylinders, i.e. the density of the N-halves. However, they are much thinner than expected.

## Views in tilt

In the Fourier-filtered electron micrograph (Fig. 1b), cylinders tilted with similar angles cluster with each other, showing the local disorder of the crystal (Fig. 3). In the Fourier space (Fig. 2), spots distribute towards higher resolutions at 1:30 and at 7:30. While, at 4:30 and 10:30, the distribution is more limited, suggesting the major direction of distortion.

**Fig. 5:**
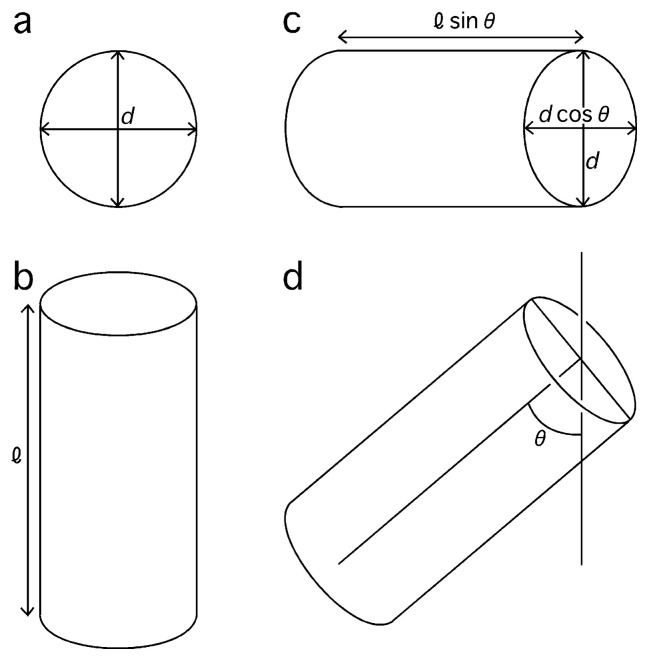
A projection (a) of a cylinder of the diameter *d* through its helical axis of length *l* (b) with no tilt, and another projection (c) with tilt ***θ*** (d).

By examining the tilted cylinders (Fig. 3), the length of the cylinders *l* (i.e. the thickness of the microcrystal along its z axis) can be determined. First, the tilt angle ***θ*** is determined by measuring the distortion of the circles circumscribing hexagons when projected (i.e. *d*cos***θ*** in comparison with *d* in Fig. 5c), and using this angle, the length *l* is determined from the projected length of cylinders *l*sin***θ*** (Fig. 5c).

In this way the *l* length was determined to be 200–300 Å (i.e. with the known pitch of the cylinder 47.4 Å, corresponding to 5–6 helical turns), using the two examples shown in Fig. 3a, b. An averaged view of a pseudo-hexagonal unit (Fig. 3c) was obtained by using 18 examples in Fig. 3a, and another average (Fig. 3d) by using 19 examples in Fig. 3b.

## Factors limiting the resolution

Currently, the Fourier spots identified (Fig. 2) do not reach 30 Å, showing the limitation of the resolution. The disorder inside the crystal appears to be the major factor for this limitation: a better microcrystal is needed.

With the magnification 66.5 K, each CCD pixel covers an area as large as 5.27 × 5.27 Å, and thus, even if an ideal microcrystal is obtained, the resolution will not be much improved: a higher magnification is required.

Problems are associated with the image processing as well, in particular, with choices for particular contrast and brightness of each image. These parameters are changed automatically by computer programs at steps. On top of this, depending on the design of a printer, an image shown on the screen is altered upon printing in a particular way: printers too change these parameters automatically. In order to better define the density of FL11, in particular, its N-half, a more objective and sophisticated method for defining these parameters will need to be introduced.
